# Nanoparticles Biosynthesized by Fungi and Yeast: A Review of Their Preparation, Properties, and Medical Applications

**DOI:** 10.3390/molecules200916540

**Published:** 2015-09-11

**Authors:** Amin Boroumand Moghaddam, Farideh Namvar, Mona Moniri, Paridah Md. Tahir, Susan Azizi, Rosfarizan Mohamad

**Affiliations:** 1 Department of Bioprocess Technology, Faculty of Biotechnology and Biomolecular Sciences, Universiti Putra Malaysia, Serdang, Selangor 43400 UPM, Malaysia; E-Mails: amin.broomandm@yahoo.com (A.B.M.); Mona_moniri6@yahoo.com (M.M.); azisusan@gmail.com (S.A.); 2 Institute of Tropical Forestry and Forest Products (INTROP), Universiti Putra Malaysia, Serdang, Selangor 43400 UPM, Malaysia; E-Mail: parida.introp@gmail.com; 3 Research Center for Animal Development Applied Biology & Department of Medicine, Mashhad Branch, Islamic Azad University, Mashhad 91735, Iran

**Keywords:** nanoparticle, fungi, yeast, apoptosis, anti-angiogenesis

## Abstract

In the field of nanotechnology, the use of various biological units instead of toxic chemicals for the reduction and stabilization of nanoparticles, has received extensive attention. Among the many possible bio resources, biologically active products from fungi and yeast represent excellent scaffolds for this purpose. Since fungi and yeast are very effective secretors of extracellular enzymes and number of species grow fast and therefore culturing and keeping them in the laboratory are very simple. They are able to produce metal nanoparticles and nanostructure via reducing enzyme intracellularly or extracellularly. The focus of this review is the application of fungi and yeast in the green synthesis of inorganic nanoparticles. Meanwhile the domain of biosynthesized nanoparticles is somewhat novel; the innovative uses in nano medicine in different areas including the delivery of drug, cancer therapy, antibacterial, biosensors, and MRI and medical imaging are reviewed. The proposed signaling pathways of nanoparticles induced apoptosis in cancerous cells and anti-angiogenesis effects also are reviewed. In this article, we provide a short summary of the present study universally on the utilization of eukaryotes like yeast and fungi in the biosynthesis of nanoparticles (NPs) and their uses.

## 1. Introduction

Nanotechnology is an innovative field which influences all aspects of human’s life [1,2]. Nanoparticles (NPs) are applied in a variety of majors such as “nanomedicine”, they are paid so much attention in this field [3].Nanoparticles can be widely classified into two groups of organic and inorganic ones. While organic nanoparticles consist of carbon nanoparticles, a number of the inorganic nanoparticles contain magnetic nanoparticles, the noble metal nanoparticles (like gold and silver) and semi-conductor nanoparticles (like titanium oxide and zinc oxide). Since inorganic nanoparticles are recently utilized as catalysts [4], semiconductors [5], optical devices, biosensors [6], encapsulation of drugs, and contrast agents, to name a few, their production has attracted great attention. In addition, as inorganic biomass nanoparticles *i.e.*, of noble metal nanoparticles (gold and silver) catering for better-quality material properties with functional flexibility, there is an increasing interest in their formation.

The most significant biomedical agents are considered as metallic nanoparticles. To synthesize the nanoparticles, silver, aluminum, gold, zinc, carbon, titanium, palladium, iron, fullerenes and copper have been regularly utilized. The Au-NPs were used in the 16th century, for both medical and staining aims [7]. As such, the development of environmentally friendly procedures via green synthesis and further biological methods is highly needed.

A number of scientists have developed an assortment of chemical and physical methods to attain such geometries which can be utilized in diverse applications. Photolithography [8], electron, ion beam lithography [9], dip pen lithography [10], micro contact printing, electrochemical synthesis and nano imprint lithography are considered as new techniques for achieving such sole geometries in nanomaterial’s. The geometries can be achieved by employing the physical approach [11]. The chemical processes begin with decreasing the metal ions to metal atoms which is pursued by controlled mass of atoms [12].

**Scheme 1:**
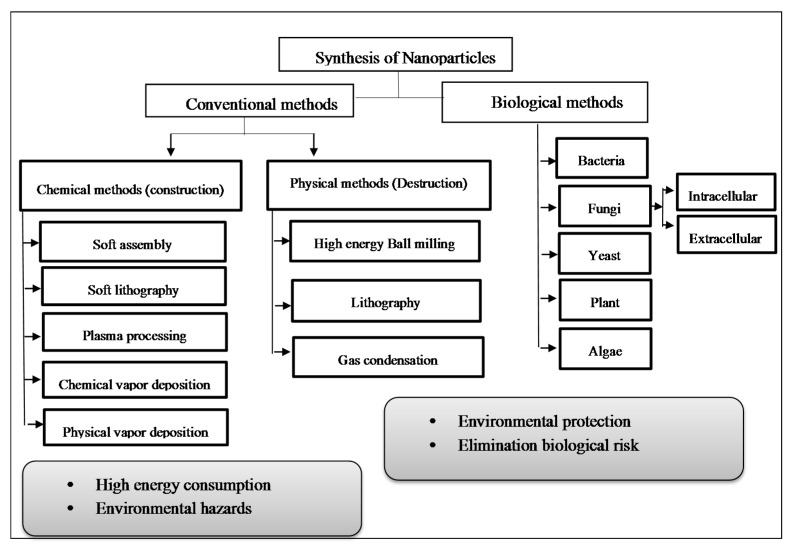
Different synthetic approaches of metallic nanoparticles.

The majority of the chemical and physical techniques employed for the synthesis of nanoparticles are very costly. Moreover, it contains the application of poisonous and dangerous chemicals responsible for different biological hazards (Scheme 1). This matter increases the necessity of developing environmentally friendly procedure by means of green synthesis and extra biological methods. In this article, we provide a summary of the present study universally on the implication of microorganisms like yeast and fungi in the biosynthesis of inorganic nanoparticles, their proposed signaling pathways and uses.

## 2. Biological Synthesis of Nanoparticles

For the synthesis of metallic nanoparticles, living extracts have been utilized by researchers. They followed easy processes such as the procedures of reducing the metal ions. In doing so, they made use of biomass extracts as a basis of extracellular or intracellular reductants.

Several molecules including carbonyl groups, terpenoids, phenolic, flavones, amines, amides, proteins, pigments, alkaloids and additional decreasing factors existing in the plant extracts and microbial cells may cause synthesis of nanoparticles [13]. In this regard, the firm mechanism of metallic nanoparticles synthesis via biomass extracts is not recognized (Table 1). To produce more tiny particles in a large scale, the biosynthesis can be effectively employed [14].It is worth mentioning that NPs which are biologically synthesized reveal increased constancy [15] and they control morphology better [16].

**Table 1:** Possible mechanism of nanoparticle biosynthesis using different sources.

Biomass	Possible Mechanism of Nanoparticle Biosynthesis	Reference
**Plant** ➢ **Leaves** ➢ **Stems** ➢ **Roots** ➢ **Shoots** ➢ **Flowers** ➢ **Barks** ➢ **Seeds**	Secondary metabolites (alkaloids, flavonoids, saponins, steroids, tannins and other nutritional compounds) acts as reducing and stabilizing agents	[17]
**Algae** ➢ **Macro algae** ➢ **Micro algae**	Polysaccharides have hydroxyl groups and other functionalities that can play important roles in both the reduction and the stabilization of nanoparticles	[18,19,20,21]
**Fungi**	Reducing enzyme intracellularly or extracellularly and the procedure of biomimetic mineralization	[22,23]
**Yeast**	Membrane bound (as well as cytosolic) oxido reductases and quinones	[24]
**Bacteria**	The microbial cell reduces metal ions by use of specific reducing enzymes like NADH-dependent reductase or nitrate dependent reductase	[25,26]

## 3. Biosynthesis of Nanoparticles by Microorganisms

It is recognized that microorganisms including bacteria, *Cyanobacteria*, *Actinomycetes*, yeast and fungi make inorganic nanoparticles such as gold, silver, calcium, silicon, iron, gypsum and lead. Because of their intrinsic potential, they produce nanoparticles, which are intra and/or extracellularly in nature [27]. However, due to extra processing phases like ultra-sonication and therapy with proper detergents, it is hard to extract the nanoparticles produced through intracellular biosynthesis [28]. As a result, screening of the microorganisms resulting in biosynthesis of nanoparticle esextracellularly is necessary [29,30]. Currently, microbial approaches in the production of nanomaterials of variable compounds are mostly restricted to metals, a few metal sulfide, and very little oxides.

All of them are confined to the microorganisms of earthy source. Culture conditions determine the biological synthesis of nanoparticles through the utilization of microorganisms and consequently, it is necessary to standardize these circumstances for the production of nanoparticles in a large scale. While strict inspection over form, size, and combination of the particles is exercised, it is recognized that many microorganisms can produce metallic nanoparticles having characteristic features similar tonanomaterial’s which are synthesized chemically [31]. It is hoped that by means of hydrolytic activity of the microorganisms, other metal oxides can also be formed. In conclusion, under moderate pressures and temperatures, nano-sized materials can be produced by microorganisms. Moreover, it is inexpensive, undemanding, effective, energy-saving, and environment-friendly to make use of microbial procedure for the production of nanomaterials [32].

## 4. Biosynthesis of Nanoparticles by Fungi

**Scheme 2:**
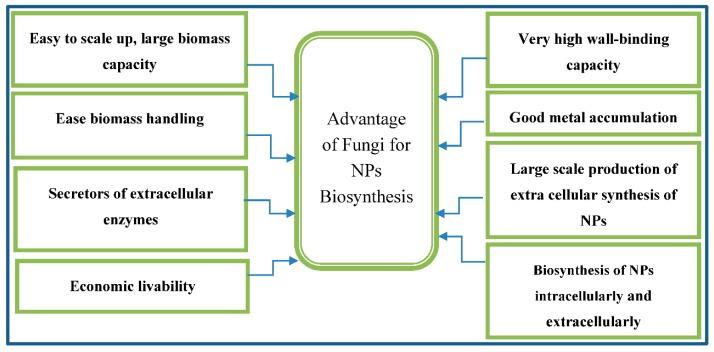
Fungi have some distinct advantages when used as bio factories for NP production.

Fungi are recognized as eukaryotic organisms that reside in various ordinary lodgings and they typically form decomposer organisms. From an anticipated sum of 1.5 million species of fungi on Earth only about 70,000 species have been recognized. The estimation of more recent data shows that according to high-through put sequencing methods, approximately 5.1 million fungal species are found [33]. It is worth mentioning that digesting extracellular food, discharging particular enzymes to hydrolyze complicated compositions into easier molecules, which are soaked up and utilized as an energy resource are the abilities of these organisms [33]. The exploration of the implication of fungi in nano biotechnology is considered important. In this regard, fungi have attracted more attention regarding the research on biological production of metallic nanoparticles due to their toleration and metal bioaccumulation capability [34].The easiness of fungi scale-up is a separate privilege of utilizing them in nanoparticle synthesis (e.g., utilizing a thin solid substrate fermentation technique). Since fungi are very effective secretors of extracellular enzymes, therefore achieving vast production of enzymes is feasible [35]. Economic livability and facility of employing biomass is another merit for the utilization of green approach mediated by fungal to synthesize metallic nanoparticles. Moreover, a number of species grow fast and therefore culturing and keeping them in the laboratory are very simple [36]. High wall-binding and intracellular metal uptake are the capacities of most fungi [37]. Fungi are able to produce metal nanoparticles/meso and nanostructure via reducing enzyme intracellularly or extracellularly and the procedure of biomimetic mineralization (Scheme 2) [22,23].

As NP processes in nanotechnology, the study of fungal species is somewhat new. One of the primary investigations of the biosynthesis of metallic NPs by means of fungi illustrates the synthesis of silver NPs extracellularly by the filamentous fungus *Verticillium* sp. [38]. For this aim, the filamentous fungus *Fusarium oxysporum* has been widely utilized species among the fungal ones identified for NP synthesis (Table 2).

In most cases, the synthesis of extracellular NPs is published, though biomass has usually exposure to metallic ion solutions [39]. First utilized fungus is reported as individual CdS NPs in addition to the formation of PbS, ZnS, and MoS_2_ NPs. A feasible sulfate diminishing enzyme-based procedure for NPs production was suggested by the existence of proteins in the aqueous solution. By utilizing the identical fungus, silver NPs were achieved which emerged separately. They can also appear through gathering with highly changing morphology in a size which ranges from of 5–50 nm [22].

In addition, in another study, the results showed that spherical silver NPs in a size range of 20–50 nm via the utilization of *F. oxysporum* was produced [23]; by comparison the results of the two mentioned studies [22,23], the discrepancy in morphology and size could be ascribed to variations in used temperature, although it appeared that the size of NPs did not depend on time [40]. Although the most frequently formed NPs are quasi-spherical ones, different morphologies can be achieved according to the metallic ion solution and incubation circumstances.

By employing *F. oxysporum*, the synthesis of NPs having different metals has been conducted [23]. Extracellular production is reported in all cases having a variety of size ranges in addition to different forms (Table 2). The decrease in the metal ions through this fungus has been related to a NADH-based reductases and a shuttle Quinone extracellular procedure [38]. 

In addition, it was discovered that different quantities of NADH made the synthesis of Au-Ag alloy NPs with diverse compounds possible [31]. Furthermore, purified α-NADPH-dependent nitrate reductase produceby*R. Stolonifer*, as well as phytochelatin, was utilized to form silver NPs in a size range of 10–25 nm successfully [41]. In this regard, Govender *et al.* suggested a mechanism to reduce biologically H_2_PtCl_6_ and PtCl_2_ into platinum NPs by means of a filtered hydrogenase enzyme from *F. oxysporum* [42].

**Table 2:** Potential fungal isolates used for the biosynthesis of metal/metal oxide nanoparticles.

Fungus Species	NPs	Localization	Size (nm)	Shape	Application	Reference
*Aspergillus fumigatus*	ZnO	Extracellular	1.2–6.8	Spherical and hexagonal	Industrial, medical and agricultural sectors	[43]
*Aspergillus oryzae*	FeCl_3_	-	10–24.6	Spherical	Agricultural, biomedical and engineering sectors	[44]
*Aspergillus tubingensis*	Ca_3_P_2_O_8_	Extracellular	28.2	Spherical	Agricultural, biomedical and engineering sectors	[45]
*Rhizopus oryzae*	Au	Cell surface	10	Nanocrystalline	Pesticides	[46]
*Rhizopus stolonifer*	Au	-	1–5	Irregularly (uniform)	-	[47]
*Aspergillus niger*	Au	Extracellular	10–20	Polydispersed	-	[48]
*Aspergillus niger*	Au	Extracellular	12.79 ± 5.61	Spherical	-	[49]
*Aureobasidium pullulans*	Au	Intracellular	29 ± 6	Spherical	-	[50]
*Colletotrichum* sp.	Au	-	20–40	Decahedral and icosahedral	-	[51]
*Fusarium semitectum*	Au	-	25	Spherical	Optoelectronics	[52]
*Fusarium oxysporum*	Au	-	2–50	Spherical, monodispersity	-	[50]
*Fusarium oxysporum*	Au	Intracellular	128 ± 70 ^a^	Aggregates	-	[11]
*Helminthosporum solani*	Au	Extracellular	2–70	Polydispersed	Anti-cancer drug	[53]
*Neurospora crassa*	Au	-	32	Spherical	-	[36]
*Penicillium brevicompactum*	Au	-	10−50	Spherical	To target cancer cells	[54]
*Verticillium* sp.	Au	Cell wall	20 ± 8	Spherical	-	[55]
*Verticillium* sp.	Au	*Cytoplsmicmembran*	20 ± 8	Quasihexagonl	-	[55]
*Verticillium luteoalbum*	Au	Intracellular	<10	Spheres and rods	-	[56]
*Cylindrocladium* *floridanu*	Au	Extracellular	19.5	Spherical	-	[57]
*Phanerochaete chrysosporium*	Au	Extracellular	10–100	Spherical	-	[58]
*Volvariella volvacea*	Au	-	20–150	Spherical	Therapeutic	[59]
*Sclerotium rolfsii*	Au	Extracellular	25	Triangles, decahedral, hexagonal and rods	-	[60]
*Fusarium oxyporum*	Au	Extracellular	8–40	Spherical and triangular	-	[61]
*Fusarium oxyporum*	Au	Extracellular	46.21	Spherical, triangular	-	[62]
*Colletotrichum* sp.	Au	Extracellular	8–40	Spherical	-	[51]
*Rhizopus stolonifer*	Au	-	1–5	Irregularly	-	[41]
*Verticillium luteoalbum*	Au	Intracellular	Various	Various	-	[63]
*Coriolis versicolor*	Au	Extra- and intracellular	20–100, 100–300	Spherical and ellipsoidal	-	[64]
*Rhizopus oryzae*	Au	-	Various	Triangular, hexagonal, pentagonal, spheroidal, sea urchin like, 2D nanowires, nanorods	-	[65]
*Aspergillus niger*	Au	-	Various	Plates, aggregates, spherical	-	[48]
*Aspergillus niger*	Au	-	Various	Nanowalls, spiral plates, spherical	-	[48]
*Aspergillus niger*	Au	-	50–500	Nanoplates	-	[48]
*Candida albicans*	Au	-	20–40, 60–80	Spherical & nonspherical	Detection of liver cancer	[66]
*Verticillum* sp.	Ag	Intracellular	25	Spherical	-	[38]
*Fusarium oxyporum*	Ag	Extracellular	5–15	Highly variable	-	[67]
*Fusarium oxyporum*	Ag	Extracellular	20–50	Spherical	Antibacterial	[23]
*Fusarium oxyporum*	Ag	-	10–25	Aggregates	-	[68]
*Aspergillus fumigatus*	Ag	-	5–25	Mostly spherical, some triangular	-	[69]
*Aspergillus niger*	Ag	Extracellular	3–30	Spherical	Antibacterial and antifungal activity	[70]
*Aspergillus fumigatus*	Ag	-	15–45	Mostly spherical	Antiviral against HIV-1	[71]
*Pleurotus sajor caju*	Ag	Extracellular	30.5	Spherical	Antibacterial activity	[72]
*Aspergillus flavus*	Ag	On cell wall surface	8.92	Spherical	-	[73]
*Aspergillus niger*	Ag	-	5–35	Spherical	Antimicrobial	[32]
*Trichoderma asperellum*	Ag	-	13–18	Nanocrystalline	Agriculture	[74]
*Volvariella volvaceae*	Ag	-	15	Spherical	Medical applications	[15]
*Penicillium fellutanum*	Ag	Extracellular	5–25	Mostly spherical	-	[75]
*Penicillium* *strain J3*	Ag	-	10–100	Mostly spherical	-	[76]
*Cladosporium cladosporioides*	Ag	-	10–100	Mostly spherical	-	[77]
*Phoma glomerata*	Ag	-	60–80	Spherical	Antibiotic	[78]
*Coriolis versicolor*	Ag	Extra- and intracellular	25–75, 444–491	Spherical	-	[79]
*Trichoderma viride*	Ag	-	5–40	Spherical, rod-like	Antibacterial activity	[80]
*Trichoderma viride*	Ag	-	2–4, 10–40, 80–100	Spherical	-	[81]
*Trichoderma viride*	Ag	-	2–4	Mostly spherical	Biosensor and bio imaging	[82]
*Trichoderma viride*	Ag	Extracellular	5–40	Spherical, rod-like	synergistic effect with antibiotics	[83]
*Amylomyces rouxii* *KSU-09*	Ag	-	5–27	Spherical	Antimicrobial	[84]
*Aspergillus clavitus*	Ag	Extracellular	550–650	-	Antimicrobial	[85]
*Aspergillus flavus* *NJP08*	Ag	-	17	Spherical	-	[86]
*Rhizopus stolonifer*	Ag	-	25–30	Quasi-spherical	-	[41]
*Aspergillus terreus CZR-1*	Ag	Extracellular	2.5	Spherical	Agriculture, Biomedical and engineering sector	[87]
*Volvariella volvaceae*	Au-Ag	Extracellular	20–150	Triangular	Medical application	[15]
*Fusarium oxyporum*	Au-Ag	Extracellular	8–14	Quasi-spherical	-	[88]
*Fusarium oxysporum*	Fe_3_O_4_	Extracellular	20–50	Irregular, quasi-spherical	-	[89]
*Verticillium* sp.	Fe_3_O_4_	Extracellular	100–400, 20–50	Cubo-octahedral, quasi-spherical	-	[89]
*Aspergillus flavus*	TiO_2_	-	62–74	Spherical	Antimicrobial	[90]
*Aspergillus flavus TFR7*	TiO_2_		12–15	Extracellular	Plant nutrient	[91]
*Fusarium oxyporum*	BT	Extracellular	4–5	Quasi-spherical	-	[92]
*Fusarium oxyporum*	Cd	Extracellular	9–15	Spherical	-	[5]
*Fusarium oxyporum*	Pt	-	70–180	Rectangular, triangular, spherical and aggregates	-	[42]
*Fusarium oxysporum* f. sp. *lycopersici*	Pt	Extra-and intracellular	10–100	Hexagonal, pentagonal, circular, squares, rectangles	-	[40]
*Fusarium* spp.	Zn	Intracellular	100–200	Irregular, some spherical	-	[93]
*Aspergillus versicolor mycelia*	Hg	Surface of mycelia	20.5 ± 1.82	Alteration	-	[94]
Fungi isolated from the soil	Zn, Mg and Ti	extracellular	Various	-	-	[95]

To synthesize metal nanoparticles successfully, a considerable amount of additional fungal species were utilized as well as *F. oxysporum* (Table 2). The use of fungal biomass and/or cell-free extract yielded the synthesis of metal NPs with different shapes and sizes [62].

Although different fungal species are utilized, diverse NPs are formed under the similar experimental circumstances. For example, while particles achieved from *Verticillium* sp. presented cubo-octahedral shapes with a size range of 100 to 400 nm magnetite, NPs produced by *F. oxysporum* had irregular form showing a total quasi-spherical morphology ranging in size from 20–50 nm [89]. As a result, the kind and condensation of biomolecules formed by each fungal species, different incubation circumstances, precursor resolutions used, and response time contribute to the type of NPs. By using the fungus *Rhizopus oryzae* in producing metallic NPs, significant results were attained. In this way, controlling the shape of gold nanoparticles at room temperature was feasible via the use of fungal extract. Therefore, NPs were produced through the manipulation of main growth factors like gold ion concentration, solution pH, and response time [63,65,96].

The possible pathogenicity to humans is the main shortcoming of the use of this organism for NP formation. The potential application of NPs formed by fungal cultures is reported by some publications; in most of these studies, the evaluation of their biological impact has been conducted. 

Moreover, the results showed the victorious inhibition of microorganisms such as bacteria and fungi by the use of silver NPs alone or together with antibiotics [78]. The antimicrobial efficiency of synthesized silver NPs via the utilization of fungal species was ascertained against bacteria [70,84] and fungal pathogens [84]. 

Other metallic NPs formed by utilizing fungi as reducing agents are less evaluated NPs; however, the nano gold-bio conjugate produced with the use of *R. oryzae* presented high antimicrobial activity against pathogenic bacteria such as *P. aeruginosa*, *E. coli*, *B. subtilis*, *S. aureus*, *Salmonella* sp., and the yeasts *S. cerevisiae* and *C. albicans* [46]. In recent years, it is proved that antimicrobial activity of fungus-interceded synthesis of TiO_2_ NPs can be anew antibacterial material [90].

When being faced with hydrous metal ions such as AuCl_4_ or Ag^+^, fungi like *Verticillium*, *Fusarium oxysporum* can form extracellular [61,67] or intracellular [38,55] metal nanoparticles. Through utilizing an entophytic fungus *Colletotrichum* sp. [51], Au nanoparticles of different morphologies were formed e.g., rods, flat sheets and triangles. This matter may result in characteristics of nanomaterial’s which can differ from those usually showing spherical shapes. With the alteration of molar proportion of the metal ions in the synthetic solutions, alloy nanoparticles with various compounds can be achieved by means of this method. This technique can also be applied for the synthesis of additional alloy or composite systems such as Au-CdS, Ag-CdS, and CdS-PbS [88]. Moreover, fungi are able to form semiconductors in addition to metallic nanoparticles. For instance, while *F. oxysporum* is subjected to hydrous CdSO_4_ solution, it’s patters sulfate-decreasing enzyme and forms extracellular CdS nanoparticles [39]. By employing the microbial procedure, metal ions (including several toxic ions) can be scuttled from ores or purification of water as well as material synthesis. Microorganisms also provide cells for size and space-constrained synthesis. Nevertheless, since not the whole minerals are produced inside the cell of microorganism and even those produced inside the cell might be influenced by the growth of the cell, so the monodispersity of the minerals may become scant. Therefore, there is a need to conduct more studies which produce higher- monodispersity nanomaterials and in a wider variety of compounds via the procedure of microorganism synthesis. In this article, we provide a short summary of the present study universally on the utilization of eukaryotes like yeast and fungi in the biosynthesis of nanoparticles (NPs) and their uses.

## 5. Production of Nanoparticles by Using Yeast

Due to the mass production of NPs as the easiness of controlling yeasts in laboratory circumstances, the synthesis of numerous enzymes and rapid growth with the use of simple nutrients, the yeast strains possess more benefits over bacteria [97]. Some studies have been conducted to investigate the synthesis of metallic nanoparticles employing the yeast. However, for this aim, through utilizing the eukaryotic systems, namely, *Candida glabrata* and *S. pombe*, one of the primary methods in employing biological material was attained [98].

The possible applicability of NPs formed by yeast have been shown by a few investigations. For the fabrication of a diode cadmium, intracellular synthesized sulfide NPs by *S. pombe* were applied, which had low-voltage operation and high forward current value. It is assumed that these properties can form the artificial structure a perfect diode [99].

In addition, to synthesize silver and gold NPs, yeast strains have been utilized. It was also reported that with an employment of silver tolerant yeast strain *MKY3*, silver nanoparticles were produced extracellularly, in which the hexagonal silver nanoparticles (2–5 nm) were synthesized in log stage of growth (Table 3). According to the different warming of the cases, the standardization and documentation of appropriate circumstance for the synthesis of bulky number of silver nanoparticles were also done [100].

**Table 3:** Nanoparticle synthesis by yeast.

Yeast	NPs	Localization	Size (nm)	Shape	Application	Reference
*Candida glabrata*	CdS	Extra- and intracellular	20 Å, 29 Å	Hexamer	Physiological	[98]
*Candida glabrata*	CdS	Intracellular	-	-	-	[101]
Yeast strain MKY3	Ag	Extracellular	2–5	Twinned or multitwinned, some hexagonal	-	[100]
*Schizosaccharomyces pombe*	Cds	Extra- and intracellular	18 Å, 29 Å	-	-	[98]
*Schizosaccharomyces pombe*	Cds	Intracellular	1–1.5	Hexagonal		[99]
*Schizosaccharomyces pombe*	Cds	Intracellular	-	-		[101]
*Pichia jadinii* (*Candida utilis*)	Au	Intracellular	-	Various	-	[63]
*Yarrowia lipolytica NCIM3589*	Au	Cell surface	Varying	Particles and plates	-	[102]
Yeast	Zr	-	-	Irregular mesoporous	Fuel cells	[103]
Yeast	Zn_3_(PO_4_)_2_	Extracellular	10–80, 80–200	Rectangular	Antirust pigment and electronic luminophore	[104]

The incubation of *Yarrowia lipolytica* cells was done with changed concentrations of chloroauric acid and formed cell-related gold NPs and nano plates (Table 3). Moreover, it is found out that the quantity of cells and the utilized salt concentrations can affect the size of NPs [102]. Similarly, to fabricate an air electrode showing outstanding electro catalytic activity for oxygen decrease (ORR), zirconium phosphate with a synthesized mesoporous figuration with the use of yeast as bio template was employed [103]. So, as bio-based templates, the synthesis of zinc phosphate nano powders was conducted with yeasts. The synthesis of Zn_3_(PO_4_)_2_ powders with butterfly similar to microstructure with a size ranging from of 10–80 nm in width and 80–200 nm in length were shown by Yan *et al.* [104].

## 6. Biomedical Applications of Green Synthesis Nanoparticles

Because of the high uses of metallic NPs in biomedical domains, a constant development is feasible in this area. Having numerous viewpoints to improve the diagnosis and treatment of human sicknesses, nano medicine is a growing field of study [105]. As fluorescent biological labels [105,106], gene and drug delivery factors [107,108], and in uses like bio detection of pathogens [109], tissue engineering [110,111] tumor demolition through heating (hyperthermia) [112], MRI contrast enhancement [113], phagokinetic investigations [114], and magnetic NPs seem to be suitable for targeted drug delivery and hyperthermia applications, discrete nanoparticles are commonly used in Nano biomedicine [115].

**Scheme 3:**
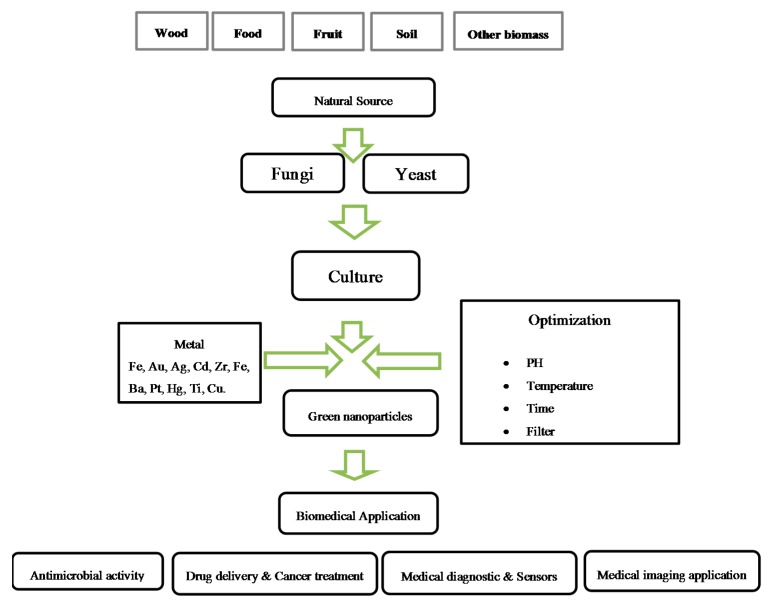
Biomedical applications of metallic nanoparticles synthesis by fungi and yeast.

The publication of numerous reviews and articles investigating the uses of nanoparticles in biomedicine has been reported [115,116]. Since the domain of biosynthesized nanoparticles is somewhat novel, investigators have already began to explore their use in different areas including the delivery of drugs, cancer therapy, gene treatment and DNA analysis, antibacterial factors, biosensors, increasing response rates, separation science, and MRI. In this article, to show these applications, a number of examples are given in Scheme 3 and described below.

### 6.1. Drug Delivery

The main concern in designing and developing new drug delivery systems is the accurate and secure delivery of the drugs to their targeted places at the proper time. The reasons is achieving a controlled release and attaining the highest therapeutic impact. In order to reach target cells, targeted Nano conveyors must pass through blood tissue obstacles [117]. Moreover, targeted Nano carriers must have contact with cytoplasmic targets through special endocytotic and transcytotic transfer mechanisms across cellular obstacles to reach targeted cells [105].

Firstly, Ag-NPs drug conveyors are able to pass through the blood-brain hindrance and the narrow epithelial joints of the skin usually hindering the delivery of drugs to the targeted place due to their tiny size. Secondly, Nano conveyors demonstrate enhanced pharmacokinetics and bio distribution of therapeutic factors and therefore reduce poison via their privileged gathering at the targeted position owing to their elevated surface area to volume proportion [118]. It is recognized that magnetic nanoparticles such as Fe_3_O_4_ (magnetite) and Fe_2_O_3_ (maghemite) are biocompatible. They have been dynamically studied for some reasons such as target cancer therapy (magnetic hyperthermia), categorization and manipulation of stem cell, trained drug delivery, gene treatment and the examination of DNA, and MRI [119].

Gold nanoparticles have been produced as potential scaffolds for the delivery of drug and gene which have complementary role over more conventional delivery carriers [120]. Novel delivery tactics can be emerged with the amalgamation of low-innate toxicity, high-surface part, steadiness, and the role of tunability which supplies them with distinctive features. Biomedical applications of chemically synthesized Au-NPs were studied before, but to our best knowledge there are no reports on the use of biosynthesized Au-NPs for drug delivery [121]. As new therapeutic factors, silver nanoparticles have been broadly utilized which their applications have also been extended as antibacterial, antifungal, antiviral and anti-inflammatory factors. In this regard, silver nanoparticles possessing anti-angiogenic potentiality formed by *Bacillus licheniformis* were investigated by Kalishwaralal *et al.* [122].

It is predicted that the number of anticancer drugs may be considerably lessened by nanoparticle-interceded targeted delivery of drugs with better property, improved efficiency, and squat toxicities. It is believed that the rising uses of nanotechnology-based therapeutics and diagnostics in clinics will be observed in the following few years. Moreover, a further significant field which nanotechnology can have a key role in individual medicine. Any special targeted treatment may not work for each sick population because of the cancer heterogeneity and progress of drug persistence. Furthermore, magnetic nanoparticles are able to be utilized for hyperthermia cancer therapy. Hyperthermia cancer therapy refers to the administration of magnetic nanoparticles into the body, especially at cancer tissue locations. Through an external magnetic field, local heating at particular locations is possible [112,123].

### 6.2. Anticancer NPs

To study biosynthesized NPs, Cytotoxicology investigations against different cancer cell lines have been reported. Some experiments utilizing synthesized Au-NPs and existing phytochemicals in grapes (*Vitis vinifera*) were published by Amarnath *et al.* [124]. These Au-NPs showed the significant similarity towards HBL-100 (human breast cancer cells), and AuNP exposure yielded HBL-100 apoptosis [124]. AuNP biosynthesis via the supernatant, live cell filtrate and biomass of the fungus *Penicillium brevicompactum* were investigated by Mishra *et al.* [54]. In another study, Jeyaraj *et al.* [125] evaluated Ag-NPs impacts on cancer cell lines.

#### 6.2.1.Proposed Signaling Pathways of Nanoparticles Induced Apoptosis

Cell proliferation inhibition is strictly linked with apoptosis. Apoptosis lead to induction of cell death by mitochondrial collapse, activation of caspase and following DNA fragmentation which is controlled by different molecules. Programed cell death or apoptosis consist of various signaling pathways including (1) ROS (reactive oxygen species)-dependent (2) Fas-dependent (3) p53-dependent and (4) p53 independent apoptosis. Some membranous proteins including Fas (death receptor) and FasL (Fas ligand), and increased cytoplasmic levels of proteins such as Smac/DIABLO, Bax (Bcl-2- associated X protein) and cytochrome c are also involved in the activation of apoptosis.

The limited literatures are available in clarifying the molecular mechanisms of nanoparticles cytotoxicity. Some studies revolve around signaling leading to cell death resulting from the cytotoxicity of these nanoparticles. For example, nanosilver induces apoptosis in NIH_3_T_3_ fibroblasts via a mitochondria-mediated mechanism of release of cytochrome c into the cytosol and the translocation of Bax to mitochondria [126]. Similarly, Vamanu *et al.* noted apoptosis mechanism to be involved in the cytotoxicity of TiO_2_ nanoparticles in human monoblastoid cell line and shed light on the mitochondria-mediated mechanism of apoptosis [127]. Park *et al.* describe the mechanisms mediating oxidative stress and apoptosis induction by titanium dioxide nanoparticles in normal human bronchial epithelial cells [128]. They observed the increased ROS levels induced by treatment of those cells with titanium dioxide nanoparticles correlated with the increased caspase-3 activity, leading to cell death [128]. Zhao *et al.* (2009) showed that titanium dioxide (TiO_2_) nanoparticles induced apoptosis in a mouse epidermal cell line (JB6 cells) in a time and dose-dependent mode. They reported remarkable mitochondrial and lysosomal membrane injury and activation of caspase-8, caspase-3, Bid and Bax, and a decrease of Bcl-2 by TiO_2_ nanoparticles [129]. The major role in TiO_2_-induced apoptosis is caspase-8/Bid signaling.

**Figure 1:**
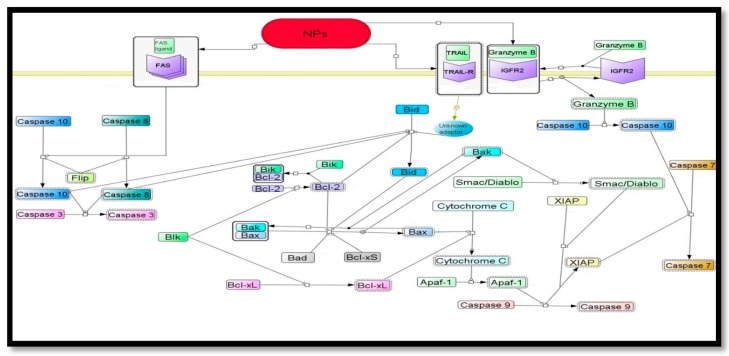
Proposed signaling pathways of nanoparticle induced apoptosis in cancerous cells. Apoptosis is induced by an apoptotic signal from NPs.

Although internalization of mesoporous silica nanoparticles did not affect the viability of human mesenchymal stem cell, it did induce a transient protein response and enhanced osteogenic signaling in these cells [130]. In another study TiO_2_ micro and nanoparticles appeared to induce apoptosis and necrosis and/or a new type of cell death mechanism that contains features of both apoptosis and necrosis in MEF cells [131]. Clearly these studies demonstrate that the mechanisms underlying the cytotoxicity of metallic nanoparticles in mammalian cell types are far from being fully understood and merit further study. Figure 1 shows proposed signaling pathways of nanoparticles induced apoptosis.

#### 6.2.2. Anti-Angiogenesis Signaling Pathways Possibly Modulated by Nanoparticle

**Figure 2:**
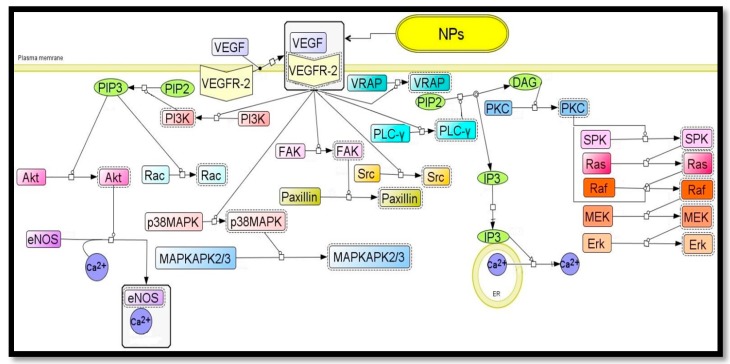
The proposed signaling pathways of nanoparticles anti-angiogenesis effects.

Angiogenesis, a physiological process involved in the growth of new blood vessels from pre-existing vessels, plays a key role in many diseases including diabetic retinopathy and cancer. The formation of new blood vessels is needed for the delivery of oxygen and nutrients to the tumor microenvironment [132]. Therefore, anti-angiogenic therapy is one of the most promising approaches to control tumor growth and metastasis. In normal and pathological angiogenesis, growth factors such as VEGF and fibroblast growth factor (FGF) are considered the major angiogenic factors that play a crucial role. The anti-angiogenic properties of gold nanoparticles clarify their effective role in treatment against progression of tumor models including ovarian cancer [133]. These NPs have also recently emerged as an attractive candidate for delivery of various payloads into their targets [125,126]. (Figure 2) shows the proposed signaling pathways of nanoparticles anti-angiogenesis effects. Baharara *et al.* demonstrated that green silver nanoparticles synthesized from *Achillea bibersteinii* (Ab. Ag-NPs) have cytotoxic effects on the endothelial cells. This Ab. Ag-NPs showed dose-dependent cytotoxicity against the endothelial cells. Another study also showed that Ag-NPs lead to indirect effects to the microcirculation in the chick embryo chorioallantoic membrane (CAM). These effects were associated with the partial preservation of the capillary diameters and connectivity happened without loss of embryo viability [134]. Ag-NPs could inhibit the vascular endothelial growth factor (VEGF) and effectively inhibited the formation of new blood micro vessels induced by VEGF in the mouse Matrigel plug assay [135,136]. Similar studies have confirmed the inhibitory effect of Ag-NPs on the vascular permeability induced by VEGF, interleukin (IL)-1β, in retinal endothelial cells [136]. The mechanism may be due to induced apoptosis that affects the proteins and enzymes with thiol groups like thioredoxin, thioredoxin peroxidase and glutathione, which are responsible for neutralizing the oxidative stress of Reactive Oxygen Species (ROS) that are largely generated by mitochondrial energy metabolism [137].

### 6.3. Antibacterial Agent

In recent years, with the outbreak and augmentation of the resistance of microorganisms to numerous antibiotics, there has been an emphasis on silver-based antiseptics. With the utilization of fungus *Trichoderma viride*, the biosynthesis of *s*ilver nanoparticles was done [83]. It was found that when exposed to a filtrate of *T. viride*, aqueous silver (Ag^+^) ions were lessened in solution, thus resulting in the production of enormously steady Ag-NPs with the size range of 5–40 nm. In addition, the nanoparticles were investigated for their augmented antimicrobial activities with a variety of antibiotics *vs.* Gram-positive and Gram-negative bacteria. The antibacterial activities of ampicillin, kanamycin, erythromycin, and chloramphenicol were augmented with the existence of Ag-NPs *vs.* test strains. The maximum increasing impact was noticed for ampicillin against test strains. The findings illustrated that the amalgamation of antibiotics associated with Ag-NPs has improved antimicrobial impacts and catered for useful understanding of the formation of novel antimicrobial factors. Dur’an *et al.* found that to avoid or reduce the disease caused by pathogenic bacteria like *Staphylococcus aureus*, extracellular formed silver nanoparticles utilizing *Fusarium oxysporum* can be integrated into textile fabrics [138].

#### Antifungal Activity

Several studies have described the bacteria *Aspergillus niger* antifungal activity of biosynthesized NPs. Gajbhiye *et al.* [139] described the antifungal properties of biosynthesized NPs against *Phoma glomerata*, *P. herbarum*, *Fusarium semitectum*, *Trichoderma* sp. and *Candida albicans* in combination with fluconazol (a triazole antifungal drug). Ag-NPs biosynthesized by another fungus, *Alternaria alternata*, increased the antifungal activity of fluconazole against all tested strains except *P. herbarum* and *F. semitectum*. In another study, to biosynthesize the Ag-NPs efficient against the bacteria *Shigella dysenteriae* type I, *Staphylococcus aureus*, *Citrobacter* sp., *E. coli*, *Pseudomonas aeruginosa* and *Bacillus subtilis*, as well as the fungi *Candida albicans* and *Fusarium oxysporum*, mycelia-free water extracts of the fungal strain *Amylomyces rouxii* were utilized [84]. Moreover, the antifungal activity of biosynthesized Au-NPs has been described. Das *et al.* [46] synthesized the Au-NPs on the fungus surface, *Rhizopusoryzae* and they showed the growth inhibition of G^−^ and G^+^ bacterial strains, in addition to the fungi *Saccharomyces cerevisiae* and *C. albicans*. Au-NPs preventing the development of *C. albicans* have also been achieved following the biosynthesis with the use of a banana peel extract [140].

### 6.4. Biosensor

Nanoparticles have appealing optical and electronic features and can be utilized in biosensor applications. Zheng *et al.* found that the yeast cells-mediated biosynthesis of Au-Ag alloy nanoparticles were used for the fabrication of a susceptible electronically chemical vanillin sensor [141]. In addition, electrochemical-based studies showed that the vanillin sensor based on Au-Ag alloy nanoparticles-modified glassy carbon electrode has the ability to increase the electro chemical response of vanillin for the minimum of five times. The oxidation climax flow of vanillin at the sensor augmented in a linear way with its condensation in the size range of 0.2–50 µM with small detection limit of 40 nM under favorable functioning circumstances. To determine the vanillin among vanilla bean and the sample of vanilla tea, this vanillin sensor was used. It is suggestive that it may be usefully applied in vanillin controlling systems. In a different investigation, according to observations showing the augmentation of the enzyme activity of GOx by Au-NPs, AuNP-based- glucose oxidase (GOx) biosensors were formed [142]. The range of linear response for the glucose biosensor is 20 µM to 0.80 mM glucose with a detection limit of 17 µM (S/N = 3). To find the glucose content in business glucose injections, this kind of biosensor was used.

### 6.5. Medical Imaging

There has been an interest in the exploration of the optical features of metallic nano crystals recently. The formation of metal NPs with different sizes, forms and dielectric features has been feasible via the integration of biosynthesis techniques. Optical characteristics connected with metallic NPs involve a low or high-refractive indicator, great clearness, new photoluminescence features photonic crystals and Plasmon resonance [143]. Moreover, Nano photonics is a domain, where the light interacts with particles more tiny than its wave-length resulting in novel phenomena, like localized surface Plasmon resonance and a size-reliant semiconductor band gap [144]. By making use of *a Trichoderma viride* filtrate, fungal-mediated Ag-NPs were formed. After laser excitation, photoluminescence measurements emissioned in the range of 320–520 nm, allowing such Ag-NPs suitable for upcoming uses of labeling and imaging. Sarkar *et al*. also conducted an identical investigation [145]. The vast contact of susceptible tissues out of the operational domain has been regarded as a significant difficulty of modern laser medicine. Therefore, by locating dyes allowing the turnable blurring of the radiation onto the surface of irradiated tissues, this difficulty can be resolved. This phenomenon is named as optical radiation limiting [146]. Two more studies investigating the cadmium telluride quantum dots (CdTe QDs) fabricated through extracellular synthesis with the use of *Saccharomyces cerevisiae* [147] and *Escherichia coli* were published by other researchers [148]. These researchers examined the NP’s size-based optical features. The results showed that CdTe QD were rather tiny, covered with protein and had high solvable ability in water. By employing UV-visible spectroscopy and spectrofluorimetry with photoluminescence emission from 488 to 551 nm, the optical features were investigated in both samples. CdTe QDs associated with folic acid were utilized *in vitro* imaging of cancer cells, and were identified to have biocompatibility in a cytotoxicity assay [148].

## 7. Conclusions

The “green” method for nanoparticle synthesis, which is rapidly replacing traditional chemical syntheses, is of great interest because of eco-friendliness, economic views, feasibility and wide range of applications in several areas such as nano medicine and catalysis medicine. Recently, various types of biological units which serve a dual role as both the reducing and stabilizing agents have been used in the synthesis of bioactive nanoparticles. As summarized in this review, biologically active products from fungi and yeast represent excellent scaffolds for this purpose. Since the domain of biosynthesized nanoparticles is somewhat novel, in this article their use in different areas including the delivery of drug, cancer therapy, gene treatment and DNA analysis, antibacterial factors, biosensors, increasing response rates, separation science, and MRI are provided.
